# Spatial Disparities in Access to Dialysis Facilities in Texas: An Analysis of End-Stage Renal Data in 1974–2020

**DOI:** 10.3390/healthcare12222284

**Published:** 2024-11-15

**Authors:** Dongeun Kim, Yongwan Chun, Daniel A. Griffith

**Affiliations:** School of Economic, Political and Policy Sciences, The University of Texas at Dallas, Richardson, TX 75080, USA; dagriffith@utdallas.edu

**Keywords:** spatial disparities, enhanced two-step floating catchment area, spatial accessibility, healthcare inequities, eigenvector spatial filtering

## Abstract

Background/Objectives: This study investigates the spatial disparities in access to dialysis facilities across Texas. The objective is to analyze how urbanization and socio-economic/demographic factors influence these disparities, with a focus on differences between urban and rural areas. Methods: The enhanced two-step floating catchment area method is employed to calculate accessibility scores to dialysis facilities across the state. Additionally, Moran eigenvector spatial filtering is utilized to analyze the influence of urbanization and socio-economic/demographic factors on accessibility disparities. Results: The Moran eigenvector spatial filtering analysis revealed a significant level of spatial autocorrelation in accessibility scores, particularly highlighting disparities between urban and rural areas. Urban regions, especially major metropolitan areas, achieved higher accessibility scores due to the dense concentration of dialysis facilities. In contrast, rural areas, notably in western and northern Texas, exhibited lower accessibility, underscoring the challenges faced by residents in these regions. The model further identified urbanization and the percentage of the elderly population as critical covariates affecting accessibility, with urban counties showing higher accessibility and elderly populations in rural areas facing significant challenges. Conclusions: These findings emphasize the importance of considering spatial dependencies in healthcare accessibility studies. They suggest the need for targeted policy interventions to address the identified disparities, particularly in underserved rural regions, to improve access to dialysis facilities for vulnerable populations.

## 1. Introduction

Kidney disease represents a significant public health challenge, with kidneys being as vital as the heart, lungs, and liver organs. In the United States (U.S.), kidney disease is among the leading causes of death, with end-stage renal disease (ESRD) requiring ongoing dialysis treatments. For patients undergoing in-center hemodialysis therapy, regular visits to dialysis centers are essential for maintaining their health and quality of life. Limited accessibility to these centers can lead to a diminished quality of life and increased mortality rates among dialysis patients [1].

Texas provides a relevant context for studying dialysis access due to its relatively large ESRD patient population. According to the ESRD Network 14 2023 Annual Report [2], this organization, which serves Texas, is the largest in the U.S., with 58.2% of its dialysis patients receiving in-center treatment, accounting for 10.2% of the national ESRD patient population in 2023. Given the scale and diversity of Texas patients, understanding the factors influencing access to dialysis facilities is crucial for addressing healthcare disparities.

This study aims to assess dialysis accessibility in Texas by exploring relationships between accessibility and various demographic, socio-economic, and geographic factors. Specifically, it examines how influencers such as urbanization, income, education, insurance status, and racial and ethnic cohort membership influence access to dialysis care. By analyzing data at both the county and ZIP code levels, this research also seeks to uncover disparities in access to dialysis facilities, providing insights into how these factors contribute to variations in accessibility across Texas.

This study further investigates differences in accessibility patterns across geographic scales, offering a detailed understanding of how these factors impact access for vulnerable populations. By focusing on spatial disparities in dialysis access, this research aims to provide insights that can help improve healthcare accessibility in Texas, particularly for underserved communities.

Previous research has highlighted considerable disparities in access to dialysis care across the U.S., often linked to geographic and socio-economic factors. Papers (e.g., [3,4,5]) found that rural patients experience longer travel times to dialysis centers compared to urban patients, contributing to healthcare inequalities. Similarly, racial and ethnic minorities, particularly Black and Hispanic/Latino populations, face higher ESRD prevalence and more significant barriers to care [6,7]. Socio-economic variables, such as income, education, and insurance status, have also been identified as contributors to these disparities [8,9].

Recent advancements in spatial accessibility research, particularly through geographical information systems (GIS) based methodologies, have deepened the understanding of healthcare disparities. Studies utilizing the enhanced two-step floating catchment area (E2SFCA) method (e.g., [3,10]) demonstrate the value of integrating geographic and demographic variables to assess healthcare access. In particular, Mercen et al. (2023) [11] emphasized the multifaceted nature of disparities, showing that neighborhood characteristics, socio-economic status, and immigration status collectively shape and groom access to dialysis facilities.

In the broader context of healthcare disparities, regression analysis has been widely used to explore inequalities [12,13,14,15,16]. Unfortunately, spatial autocorrelation is generally prevalent but not well accounted for in these analyses, which have shied away from spatial regression. As a geographical phenomenon, accessibility can potentially show a significant level of spatial dependence (e.g., [17]). When spatial autocorrelation is not successfully incorporated in a regression model specification, the regression results become unreliable, a well-documented misspecification outcome (e.g., [18]). This paper extends regression modeling practice using a Moran eigenvector spatial filtering (MESF) model, which furnishes a flexible approach to account for spatial autocorrelation in georeferenced data [19].

The analyses summarized in this study using MESF provide a detailed examination of factors influencing disparities in dialysis access across Texas. By integrating advanced spatial and more traditional statistical methods, this research seeks to uncover underlying patterns and offer actionable recommendations for addressing inequities in kidney dialysis access.

## 2. Study Area and Data Sources

**Figure 1:**
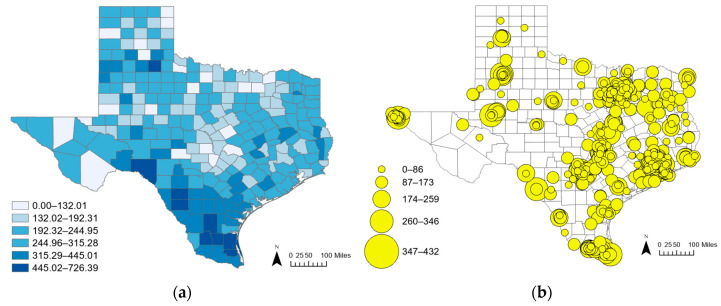
Rate of patients per 100,000 population by county (**a**) and the capacities of dialysis facilities (**b**) in Texas.

Data sources for this investigation include the National Institute of Diabetes and Digestive and Kidney Diseases’ U.S. Renal Data System (USRDS) for patient counts; the Centers for Medicare & Medicaid Services (CMS) for dialysis facility locations and capacities, including the number of dialysis machines available at each center and facility operating times; and socio-economic/demographic data from the U.S. Census Bureau [20,21,22]. The analyses were conducted at the county level for the entire state and the ZIP code level for large metropolitan areas, focusing on the following three key regions: Dallas–Fort Worth, Austin–San Antonio, and Houston. These are the three most populous metropolitan areas in Texas. Nationally, in 2024, Houston ranks fourth, Austin–San Antonio ranks seventh, and Dallas–Fort Worth ranks ninth in terms of population size. Together, they account for 66.03% of Texas’s ESRD patients—with 25.09% residing in Houston, 24.91% in Dallas–Fort Worth, and 16.03% in Austin–San Antonio [2]. A total of 751 dialysis facilities are distributed across the state. The centroids of the 254 counties are used to measure distances. The facilities within 50-mile buffered areas from the Texas boundaries were considered in the county-level analysis, and 30-mile buffered areas were considered in the ZIP-code-level analysis because the Children with Special Health Care Needs Program (CSHCN) Provider Procedures Manual of the Texas Health and Human Services Department suggests that out-of-state dialysis facilities must be located within 50 miles of the state border [23]. Figure 1 portrays the juxtaposition of a choropleth map illustrating the rate of patients per 100,000 population by county alongside a proportional circle map displaying the locations and capacities of dialysis facilities, where the size of the circles indicates facility capacity. Many of these outlets are concentrated in the three large metropolitan areas where the analysis was conducted, with additional clusters identified in some counties around other Texas cities, such as El Paso, Corpus Christi, Lubbock, Laredo, Amarillo, and Brownsville.

**Figure 2:**
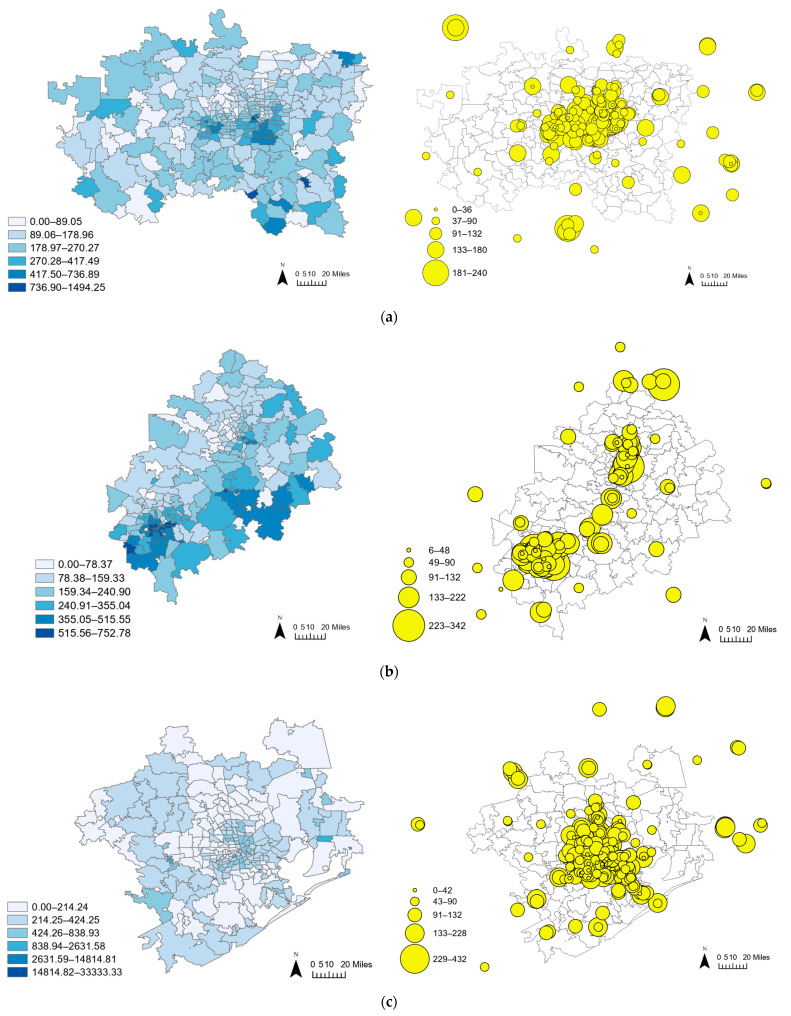
Rate of patients per 100,000 population by ZIP code (**left**) and the capacities of dialysis facilities (**right**) in each metropolitan area. (**a**) Dallas–Fort Worth area. (**b**) Austin–San Antonio area. (**c**) Houston area.

This paper also investigates the finer ZIP-code-level geographic resolution data for the three large metropolitan areas, as shown in Figure 2. The Dallas–Fort Worth area comprises 344 ZIP codes with 208 dialysis facilities, predominantly concentrated around its central urban areas and along its major transportation arteries. The Austin–San Antonio area encompasses 205 ZIP codes with 124 facilities, showing a similar pattern with facilities clustered in central locations and along key highways, indicating a strategic placement to serve densely populated regions. In contrast, with 264 ZIP codes and 248 facilities, the Houston area displays a broader distribution with a noticeable concentration in the inner city and along key transportation routes.

## 3. Methodology

The E2SFCA technique, an enhanced approach to the 2SFCA has been widely used in healthcare accessibility research. Although 2SFCA is a prevalent tool in healthcare work to assess the spatial distribution and accessibility of medical services within a given population [24], the E2SFCA extends it by incorporating a Gaussian function to assign distance-based weights when evaluating care facility accessibility. This adjustment allows for varying weights within the same catchment area, offering a detailed understanding of accessibility [25]. In contrast to traditional methods that rely solely on straight-line or network distances, the E2SFCA approach provides a more thorough and accurate picture of accessibility. It is distinguished by its robust approach to healthcare accessibility measurement, integrating both distance decay effects and travel time catchments, hence ensuring a comprehensive and precise assessment [10].

The Gaussian formula serves as a distance decay function, assigning weights to each dialysis facility based on its proximity to a target area. This function can be expressed as in Equation (1). Facilities closer to a target area receive higher weights, indicating greater accessibility, while those further away receive lower weights. This differentiation better reflects that accessibility generally decreases as travel distance increases. By incorporating this weighted approach, an improved estimate of the true variability in healthcare accessibility can be captured, providing more precise insights into the equitable distribution of dialysis services across different regions. Equation (1) describes this conceptualization:

(1) $$G\left(t_{kj},t_{0}\right)=\left\{\begin{array}{c} \frac{e^{-\left(\frac{1}{2}\right)\;\times \;{\left(\frac{t_{kj}}{t_{0}}\right)}^{2}-e^{-(\frac{1}{2})}}}{1-e^{-(\frac{1}{2})}}\\ 0,\;t_{kj}>t_{0} \end{array}\right.,t_{kj}\leq t_{0}$$

where

$t_{kj}$: the network distance between county/ZIP code centroid k and center j
$t_{0}$: a certain posited threshold travel distance

Threshold distances are set based on Stephens et al. (2013) [5], which highlights differences in travel distances between urban and rural dialysis patients in the U.S. Urban patients typically travel an average of 6.8 miles, whereas rural patients tend to travel substantially farther, averaging 28.83 miles to reach alternative dialysis facilities. In addition, the travel distances between patients and dialysis centers are often found to follow a chi-squared distribution [26]. To capture the vast majority of patients, threshold distances encompassing 99% of the area in the tail of the chi-squared distribution were chosen, whose critical values are 49.37 and 18.15, respectively. Consequently, a relaxed threshold of 50 miles was set for county-level analysis covering both urban and rural areas, and a tighter threshold of 20 miles for ZIP-code-level analysis covering urbanized metropolitan areas.

With the Gaussian function, the dialysis service supply–demand ratio was calculated with Equation (2), which quantifies the facility service capacity relative to the patient population for each area. A higher ratio suggests greater accessibility, meaning there is sufficient capacity for dialysis services relative to the affiliated patient population. To determine the supply measurement per facility, the number of patients that each facility can accommodate weekly was considered. The average in-center treatment time is 3 to 5 h, and end-stage patients typically take their treatments three times per week. Based upon this information as input, individual dialysis service supply–demand ratios may be calculated with Equation (2) and then accumulated as accessibility scores as Equation (3):

(2) $$R_{j}=\frac{S_{j}}{\sum_{k\in \{t_{kj}\leq t_{0}\}}G\left(t_{kj},t_{0}\right)D_{k}}$$

(3) $$AMSE_{k}=\sum_{j\in \{t_{kj}\leq t_{0}\}}G\left(t_{kj},t_{0}\right)R_{j}$$

where

$R_{j}$: dialysis service supply-demand ratio in center j
$S_{j}$: capacity of the center j
$D_{k}$: the number of patients in county/ZIP code centroid k
$t_{kj}$: the network distance between county/ZIP code centroid k and center j
$G(t_{kj},t_{0})$: the distance weight for the threshold travel distance calculated with the Gaussian function, capturing the distance decay of access to center j, and $0<t_{kj}\leq t_{0}$
${AMSE}_{k}$: the accessibility score assigned to the county/ZIP code centroid k
$t_{0}$: a certain posited threshold travel distance

The dialysis service accessibility scores, as determined by the E2SFCA method, are further analyzed in conjunction with urbanization and socio-economic/demographic variables using MESF to identify the key factors that are significantly associated with spatial disparities. MESF is a spatial statistical technique employed to model spatial dependencies in data and identify underlying spatial patterns or structures, often referred to as spatial effects, within the study area [19,27]. Specifically, an eigenvector spatial filter (ESF) constructed with MESF captures spatial autocorrelation by incorporating selected eigenvectors from a modified spatial weights matrix into a regression model as new independent variables, thus accounting for spatial autocorrelation effects. This method is particularly beneficial when variables are suspected to have strong spatial dependence among areal units, as observed in the data analyses of this research.

The MESF method begins with the spectral decomposition of a transformed spatial weight matrix C, as expressed in Equation (4):

(4) $$MCM=E\Lambda E^{T}$$

where $M=(I-\frac{11^{T}}{n})$ denotes the projection matrix that centers the data; here **I** denotes an n × n identity matrix, 1 denotes an n× 1 vector of ones, and superscript T denotes the matrix transpose operator. C denotes a spatial weights matrix, E denotes a matrix having *n* column eigenvectors, and Λ denotes the diagonal matrix having eigenvalues corresponding in order to the eigenvectors in **E**.

The MESF model specification with linear regression can be expressed as Equation (5):

(5) $$Y=X\beta_{X}+E_{k}\beta_{k}+\epsilon$$

where

Y: the dependent variable
X: an n×(p+1) matrix containing covariates
$E_{k}$: an n×k matrix containing k selected eigenvectors from the full set of the eigenvectors, E, with $E_{k}\beta_{k}$ being the constructed ESF
$\beta_{k}$: the corresponding k×1 vector of regression parameters for the eigenvectors
ϵ: the error term, assumed to be independent and normally distributed

Previous research highlights factors contributing to disparities in access to dialysis facilities, including socio-economic status, insurance coverage, poverty level, and age, race, and ethnicity [28,29]. One key determinant of healthcare access is urbanization because urban areas typically have a higher concentration of healthcare facilities, including dialysis centers, due to denser populations and better infrastructure. This urban–rural divide often results in significant disparity differentials, with rural areas being underserved. Understanding the impact of urbanization is therefore crucial in addressing inequalities in dialysis access.

Socio-economic indicators, such as median household income and poverty levels, are also critical. Higher income allows individuals to afford better healthcare services and insurance, while those living in poverty may face financial barriers, limited transportation, and fewer healthcare facilities [11]. Educational attainment, measured by high school graduation rates, is linked to health literacy, which affects individuals’ ability to navigate a healthcare system. Studies show that lower levels of education correlate with poorer health outcomes and shorter life expectancy, further compounding disparities [30].

The prevalence of uninsured individuals is another significant factor, as those without insurance often delay necessary treatments due to cost concerns, exacerbating disparities in access to essential services like dialysis [11]. Demographic factors also illuminate disparities in healthcare access. The percentage of a population aged 65 or older is important, as older adults generally have greater healthcare needs, including dialysis. Although kidney function typically declines with age [31], some studies suggest that renal function in the elderly remains well-preserved unless disrupted by extreme conditions [32].

Finally, the presence of racial and ethnic minorities—such as Asian, Hispanic/Latino, and Black communities—further highlights healthcare disparities. These groups often face systemic barriers, including discrimination and limited healthcare resources, which hinder their access to necessary medical services [29].

## 4. Results

**Figure 3:**
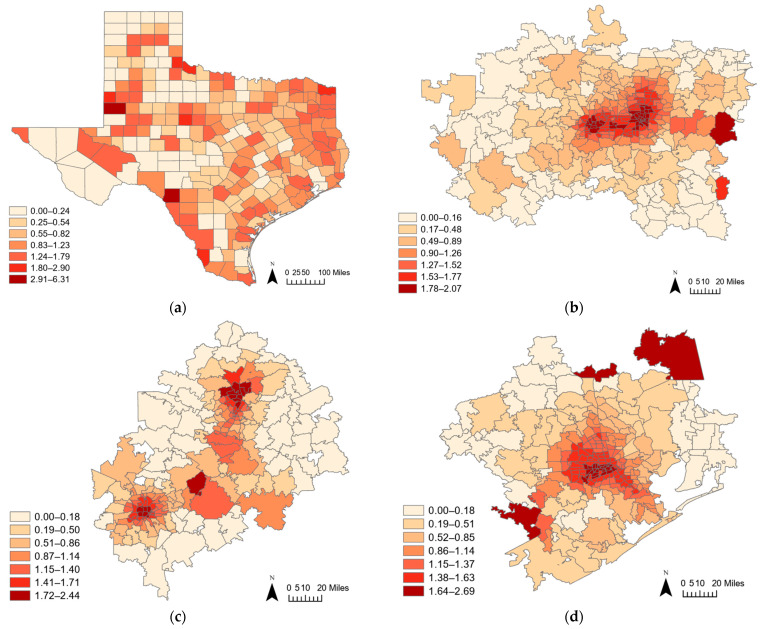
Geographic landscape accessibility scores by Texas counties (**a**) and Dallas–Fort Worth (**b**), Austin–San Antonio (**c**), and Houston (**d**) ZIP codes.

Figure 3a presents the accessibility scores for each Texas county, with dark colors indicating high scores and light colors representing low scores. The results show distinct patterns across the state. High accessibility scores primarily concentrate in metropolitan areas and more populous counties, particularly around urban centers such as Dallas–Fort Worth, Houston, and the Austin–San Antonio metropolis, where the densities of dialysis facilities are high. Additionally, relatively high scores appear along the Texas–Mexico border and East Texas. These areas, including counties such as Laredo and regions close to New Mexico, also show higher patient rates, as illustrated by the Figure 1a map. This correlation between high patient rates and elevated accessibility scores suggests that these border regions, despite being less urbanized than major metropolitan areas, still have a significant concentration of dialysis services relative to their population needs.

Conversely, counties with low accessibility scores tend to be more rural and less densely populated, often located in the western and northern regions of Texas, where the number of facilities is limited. This spatial distribution highlights the strong association between high accessibility scores and the availability of multiple facilities in populous urban areas, underscoring the disparities in access between urban and rural counties. This pattern suggests that while urban areas benefit from a higher density of services, rural regions, particularly in western and northern Texas, face significant challenges in providing adequate dialysis care, contributing to ongoing healthcare access disparities in these regions.

Figure 3b shows that the central and the eastern parts of the Dallas–Fort Worth area exhibit high accessibility scores, as indicated by dark red on that map. These areas, particularly those in the urban core, demonstrate a concentration of healthcare facilities, which is reflected in the relatively high scores compared to the surrounding localities. This map underscores the distribution of healthcare resources, with the central urban centers benefiting from the presence of several major healthcare institutions and a well-developed transportation network, which enhances accessibility (e.g., [1]). Furthermore, the considerable number of healthcare providers in these urban centers fosters a competitive environment, potentially leading to improved service delivery and greater attention to patient needs (e.g., [7]). As healthcare resources align with the growing demand in this densely populated metropolitan region, the complexity of ensuring that residents can effectively navigate their healthcare journey becomes evident (e.g., [6]).

The central and southern parts of the Austin–San Antonio conurbation have high accessibility scores (Figure 3c). These areas correspond to locations with a dense concentration of healthcare facilities, particularly urban centers. The map clearly illustrates that the highest accessibility is found in the central urban core, which has a rapid population growth driven by economic opportunities and a vibrant cultural scene. Additionally, bus service along Interstate 35 (I-35) that connects San Antonio and Austin further enhances access, allowing residents from various socio-economic backgrounds to reach these healthcare facilities more easily. The relatively high accessibility scores in these regions not only reflect the geographic distribution of healthcare facilities, but also may accentuate the influence of socio-economic factors that empower residents to utilize available services.

The central and northeastern parts of the Houston area exhibit high accessibility scores. These places have a dense urban concentration, where more healthcare facilities are located. Figure 3d highlights the central urban core as particularly well-served by healthcare resources, reflecting the presence of numerous facilities within this densely populated area. This finding is especially relevant given the area’s large and culturally diverse population, which is identified as a factor for improvements in healthcare infrastructure [28]. The Texas Medical Center and other healthcare institutions in this region substantially contribute to the high accessibility scores observed. Although the relatively high magnitudes of these scores are noteworthy, a closer examination of this map also reveals areas on the outskirts with lower accessibility, stressing the importance of addressing health disparities in these regions.

The accessibility maps provide valuable insights into the spatial distribution of healthcare facilities across Texas at both the county and ZIP code levels. The county-level result presents varying levels of accessibility across the entire state at a coarse geographic resolution. On the one hand, although this more aggregated result helps identify general patterns and disparities across the state, it can also obscure finer details, particularly in densely populated urban areas where healthcare facilities are more concentrated. On the other hand, the ZIP-code-level results for the Dallas–Fort Worth area, the Austin–San Antonio area, and the Houston area offer a more detailed view, showcasing considerable variations in accessibility within these urban contexts.

Differences between the county-level and the ZIP-code-level analyses spotlight the importance of scale in spatial analysis. Although the county-level result map provides a useful overview of general accessibility patterns across the state, the finer granular ZIP-code-level analysis reveals disparities within urban areas that might be overlooked at a broader scale. For example, central urban areas consistently show high accessibility scores, reflecting the concentration of healthcare facilities, whereas accessibility tends to decrease toward hinterland outskirts, with details more apparent at the finer scale. This multi-scalar approach helps bolster the understanding of healthcare accessibility, ensuring that both broad patterns and local variations are considered in analysis and policy-making.

Table 1 tabulates the regression results for accessibility at the county level with urbanization and socio-demographic variables. Compared to the standard aspatial linear regression specification, the MESF model yields a higher *R*^2^ value, signifying a better explanatory power and model fit for the observed data. Additionally, the ESF model addresses and accounts for spatial autocorrelation by incorporating the selected eigenvectors. That is, the spatial model containing an ESF successfully eliminates spatial autocorrelation in regression residuals.

The results indicate that counties with a high percentage of urban residents tend to have high accessibility scores for dialysis facilities. This finding suggests that urban areas are better equipped with healthcare services compared to their rural counterparts. Conversely, a significant inverse relationship exists between the elderly population rate and accessibility scores. This trend can be explained by the concentration of dialysis centers in urban areas, while the elderly population is more evenly distributed across urban, suburban, and rural regions. This finding highlights systemic inequalities in healthcare access, particularly for older adults who may face mobility challenges and have greater healthcare needs. These results are consistent with studies that emphasize the barriers to healthcare faced by older adults in rural areas, which often lack adequate healthcare infrastructure [33].

The results also show a negative correlation with high school graduation rates, suggesting that areas with low educational attainment may encounter heightened barriers in accessing essential healthcare services. This finding aligns with the broader public health literature, indicating that educational disparities significantly influence health outcomes and access to care, as individuals with low educational attainment often have reduced health literacy and fewer economic resources to seek necessary health services [34]. Race variables are not found to be significant in this analysis, contrasting with findings in other studies that have highlighted racial disparities as a key determinant of healthcare access [7,33]. This discrepancy may suggest that, at the Texas county scale level, educational attainment and socio-economic factors are more influential in shaping access to dialysis facilities than race, or it could reflect limitations in capturing the full impact of race on healthcare accessibility within this specific scale of analysis.

Table 2 presents the regression results for the Dallas–Fort Worth area. The MESF model’s *R*^2^ value and the corresponding Moran’s Coefficient indicate strong explanatory power and effective handling of spatial autocorrelation, ensuring reliable results. This spatial model highlights the positive association between urban population rates and accessibility scores, affirming that urbanization is a significant factor in determining access to healthcare services in the metropolitan area. Interestingly, the elderly population rate also shows a significant positive relationship with accessibility, suggesting that areas with a high proportion of elderly residents may have better access to dialysis facilities in this metropolitan region.

Another notable finding from the MESF modally exercise is the significant negative correlation between high school graduation rates and accessibility scores, suggesting that areas with high educational attainment may have lower access to dialysis facilities. This could be explained by the fact that middle-class households tend to reside in suburban areas in a metropolitan region. The uninsured population rate is significantly and negatively associated with accessibility, which may resonate with the critical impact of insurance coverage on access to essential healthcare services. Contrasted with the county-level result, the MESF model detects significant positive associations between accessibility scores for both the Hispanic/Latino populations and the Black population rate, indicating that areas with high concentrations of these minority populations tend to have better access to dialysis facilities. However, these covariations may only reflect the spatial patterns in facility placements in more concentrated urban centers, and further investigations are needed to better understand the implications for such subgroups, especially given contrasting findings in the healthcare access literature.

Results for the Austin–San Antonio metropolis (Table 3) are similar to the statewide county-level results. That is, accessibility scores are positively associated with urban population rates and negatively associated with elderly population rates and high school graduation rates. The Table 4 tabulations show that in the Houston area, there is a positive association between accessibility scores and both the Asian and Hispanic/Latino population rates. This consequence may indicate the effectiveness of community-centric health initiatives or resources that facilitate access for these populations [4,8], highlighting the importance of cultural competence and tailored healthcare delivery. The elderly population and high school graduate rates are not significant, which differs from the findings for the other metropolitan areas as well as the county-level data.

In summary, both the county- and ZIP-code-level analyses indicate a strong association between urbanization and accessibility scores. However, the results differ between these two scales, with the finer-scale ZIP code analysis unveiling disparities within individual local urban areas. These insights are crucial for guiding policy initiatives to reduce disparities and improve access to essential healthcare services, particularly for at-risk populations, by addressing regional patterns and localized challenges.

## 5. Discussion

This paper explores the accessibility of dialysis facilities in Texas, focusing on research questions that address disparities in access across different geographic areas and population cohorts. In addition, accessibility to dialysis facilities is examined in relation to urbanization and socio-economic/demographic factors. Additionally, it summarizes an analysis of accessibility at two different spatial scale levels (i.e., county and ZIP code) to examine potential scale effects on differences in accessibility.

Findings highlight significant disparities in access to kidney dialysis facilities across Texas, ones that are associated with urbanization, demographic, and socio-economic conditions. County level results indicate that urban areas, including the major metropolitan regions, exhibit high accessibility scores with intense concentrations of dialysis facilities and healthcare infrastructure in those areas. However, despite these higher accessibility scores, access is not uniformly experienced across all demographic groups, especially when analyzing data at the finer ZIP code level.

The analysis presented here also illuminates challenges faced by underserved and low-population areas characterized by fewer facilities and resources. Even within metropolitan areas with high overall accessibility scores, many neighborhoods beyond the administrative boundaries of large cities have limited access to care. Factors such as transportation barriers and inadequate healthcare outreach can exacerbate challenges faced by residents in these hinterlands [35]. Identifying and addressing these underserved communities is crucial for policy initiatives aimed at improving access to kidney dialysis care, particularly because these areas are often overlooked in broader analyses.

This study also reinforces the significant impact of socio-economic/demographic factors on dialysis accessibility. The negative correlation between high school graduation rates and accessibility scores, as well as the strong association between uninsured populations and reduced accessibility, illustrates systemic inequalities within Texas’s healthcare landscape. This deduction aligns with existing literature emphasizing the relationship between educational attainment, health literacy, and access to healthcare [34]. Additionally, an inverse relationship between the elderly population rate and accessibility highlights the unique challenges faced by older adults, particularly in rural areas with inadequate healthcare infrastructure.

The examination of scale effects reported here reveals that while county-level analyses might provide important insight for an entire state, ZIP-code-level analyses can uncover changes based on finer scale covariations that impact accessibility, particularly in relation to race and ethnicity. The significance of racial and demographic variables varies by scale, suggesting a potential for localized characteristics to play a critical role in healthcare service accessibility. For example, the increased accessibility for certain racial groups in metropolitan regions may be due to culturally competent healthcare initiatives, highlighting the need for targeted approaches within diverse communities to address specific access challenges.

Ultimately, while metropolitan areas in Texas exhibit high accessibility scores, this achievement does not necessarily indicate an equitably distributed network of kidney dialysis facilities. Rather, contextualizing these findings within the broader socio-economic landscape of Texas is essential. Urban centers may show promise in terms of resource availability, but further investigation into less accessible rural areas and the unique challenges they face—such as transportation barriers, fewer healthcare facilities, and restricted public health outreach—is crucial to ensuring equitable dialysis access across the state [1].

## 6. Conclusions

In conclusion, this analysis illustrates that accessibility to kidney dialysis care in Texas is influenced by a complex interplay of geographic, demographic, and socio-economic factors. Although urban centers demonstrate higher resource availability, persistent disparities necessitate further investigation into the unique barriers faced by underserved populations. Future research should focus on health outcomes related to variations in access, as well as explore historical trends in dialysis facility distribution, which may inform a comprehension of current disparities. Addressing these complexities is vital for developing targeted policy interventions that can effectively reduce disparities in kidney dialysis access across Texas, ultimately improving health outcomes for vulnerable populations and striving toward a more equitable healthcare system for the entire population across the state.

## Figures and Tables

**Table 1:** Results of linear and MESF regression for Texas, county-level data.

Variables	Linear Regression		MESF	
	Estimate	Pr (>|t|)	Estimate	Pr (>|t|)
Urban population rate	0.4956	0.0012 ***	0.4630	0.0011 ***
Age 65 ≥ population rate	−2.5454	0.0123 **	−2.0310	0.0339 **
High school graduation rate	−0.6796	0.3880	−1.4613	0.0446 **
Uninsured population rate	−2.0790	0.0293 **	−0.2170	0.8128
Hispanic/Latino population rate	−0.1032	0.1675	−0.1057	0.1207
Black population rate	−0.1110	0.0559	−0.0984	0.0597
Asian population rate	−0.0003	0.9127	0.0001	0.9861
Median income in the past 12 months	−0.5598	0.1897	−0.0011	0.9978
Below the poverty level population rate	0.5827	0.7063	1.0301	0.4907
	*R*^2^: 0.4282 MC of residuals: −0.015 (*p*-value: 0.031)		*R*^2^: 0.6517 MC of residuals: 0.203 (*p*-value: <0.001)	

Note: *** and ** indicate the 99% and 95% significant levels.

**Table 2:** Results of linear and MESF regression for the Dallas–Fort Worth metroplex, ZIP-code-level data.

Variables	Linear Regression		MESF	
	Estimate	Pr (>|t|)	Estimate	Pr (>|t|)
Urban population rate	0.8321	0.0000 ***	0.2704	0.0001 ***
Age 65 ≥ population rate	0.0404	0.8627	0.2960	0.0351 **
High school graduation rate	−0.1731	0.4210	−0.5114	0.0001 ***
Uninsured population rate	−0.1012	0.8213	−0.7368	0.0047 ***
Hispanic/Latino population rate	0.4325	0.0726	0.4225	0.0047 ***
Black population rate	0.6016	0.0049 ***	0.4422	0.0103 **
Asian population rate	0.0984	0.0050 ***	0.0350	0.1168
Median income in the past 12 months	−0.0478	0.1926	−0.0018	0.9328
Below the poverty level population rate	0.8139	0.0427 **	0.1650	0.5025
	*R*^2^: 0.6342 MC of residuals: 0.517 (*p*-value: <0.001)		*R*^2^: 0.9150 MC of residuals: −0.175 (*p*-value: 0.933)	

Note: *** and ** indicate the 99% and 95% significant levels.

**Table 3:** Results of linear and MESF regression for the Austin–San Antonio conurbation, ZIP-code-level data.

Variables	Linear Regression		MESF	
	Estimate	Pr (>|t|)	Estimate	Pr (>|t|)
Urban population rate	1.0580	0.0000 ***	0.3198	0.0000 ***
Age 65 ≥ population rate	−0.4945	0.0336 **	−0.4486	0.0002 ***
High school graduation rate	0.9104	0.0043 ***	0.3579	0.0276 **
Uninsured population rate	−0.0275	0.9694	0.2613	0.5143
Hispanic/Latino population rate	0.2790	0.1824	0.0743	0.6157
Black population rate	−0.2178	0.0052 ***	−0.0632	0.1452
Asian population rate	0.049	0.4677	0.0022	0.9524
Median income in the past 12 months	−0.1317	0.0261 **	−0.0216	0.4877
Below the poverty level population rate	1.4887	0.0020 ***	0.2156	0.4416
	*R*^2^: 0.5923 MC of residuals: 0.464 (*p*-value < 0.001)		*R*^2^: 0.9420 MC of residuals: −0.164 (*p*-value: 0.240)	

Note: *** and ** indicate the 99% and 95% significant levels.

**Table 4:** Results of linear and MESF regression for the Houston metropolitan region, ZIP-code-level data.

Variables	Linear Regression		MESF	
	Estimate	Pr (>|t|)	Estimate	Pr (>|t|)
Urban population rate	0.6186	0.0000 ***	0.1910	0.0340 **
Age 65 ≥ population rate	0.0173	0.8947	−0.4486	0.8415
High school graduation rate	−0.0008	0.9963	0.3579	0.3259
Uninsured population rate	0.0177	0.9615	0.2613	0.0585 *
Hispanic/Latino population rate	0.4031	0.0543 *	0.6451	0.0003 ***
Black population rate	0.4237	0.0105 **	0.2099	0.1313
Asian population rate	0.1811	0.0000 ***	0.1572	0.0000 ***
Median income in the past 12 months	−0.0096	0.7756	0.0240	0.3164
Below the poverty level population rate	0.3109	0.4119	−0.1935	0.5043
	*R*^2^: 0.5775 MC of residuals: 0.382 (*p*-value < 0.001)		*R*^2^: 0.8351 MC of residuals: −0.150 (*p*-value: 0.873)	

Note: ***, **, and * indicate the 99%, 95%, and 90% significant levels.

## Data Availability

The data are not publicly available due to privacy or ethical restrictions. The data reported here have been supplied by the U.S. Renal Data System (USRDS). The interpretation and reporting of these data are the responsibility of the author(s) and in no way should be seen as an official policy or interpretation of the U.S. government.
